# Involvement of the P2X7 Purinergic Receptor in Colonic Motor Dysfunction Associated with Bowel Inflammation in Rats

**DOI:** 10.1371/journal.pone.0116253

**Published:** 2014-12-30

**Authors:** Luca Antonioli, Maria Cecilia Giron, Rocchina Colucci, Carolina Pellegrini, Deborah Sacco, Valentina Caputi, Genny Orso, Marco Tuccori, Carmelo Scarpignato, Corrado Blandizzi, Matteo Fornai

**Affiliations:** 1 Division of Pharmacology and Chemotherapy, Department of Clinical and Experimental Medicine, University of Pisa, Pisa, Italy; 2 Department of Pharmaceutical and Pharmacological Sciences, University of Padova, Padova, Italy; 3 Scientific Institute IRCCS Eugenio Medea, Conegliano, Treviso, Italy; 4 Laboratory of Clinical Pharmacology, University of Parma, Parma, Italy; University Paris Sud, France

## Abstract

**Background and Purpose:**

Recent evidence indicates an involvement of P2X7 purinergic receptor (P2X7R) in the fine tuning of immune functions, as well as in driving enteric neuron apoptosis under intestinal inflammation. However, the participation of this receptor in the regulation of enteric neuromuscular functions remains undetermined. This study was aimed at investigating the role of P2X7Rs in the control of colonic motility in experimental colitis.

**Experimental Approach:**

Colitis was induced in rats by 2,4-dinitrobenzenesulfonic acid. P2X7R distribution was examined by immunofluorescence analysis. The effects of A804598 (selective P2X7R antagonist) and BzATP (P2X7R agonist) were tested on contractions of longitudinal smooth muscle evoked by electrical stimulation or by carbachol in the presence of tetrodotoxin.

**Key Results:**

P2X7Rs were predominantly located in myenteric neurons, but, in the presence of colitis, their expression increased in the neuromuscular layer. In normal preparations, A804598 elicited a negligible increase in electrically induced contractions, while a significant enhancement was recorded in inflamed tissues. In the presence of N^ω^-propyl-L-arginine (NPA, neuronal nitric oxide synthase inhibitor) the A804598 effects were lost. P2X7R stimulation with BzATP did not significantly affect electrical-induced contractions in normal colon, while a marked reduction was recorded under inflammation. The inhibitory effect of BzATP was antagonized by A804598, and it was also markedly blunted by NPA. Both P2X7R ligands did not affect carbachol-induced contractions.

**Conclusions and Implications:**

The purinergic system contributes to functional neuromuscular changes associated with bowel inflammation via P2X7Rs, which modulate the activity of excitatory cholinergic nerves through a facilitatory control on inhibitory nitrergic pathways.

## Introduction

Inflammatory bowel diseases (IBDs) include a wide spectrum of disorders, characterized by chronic or relapsing immune activation and inflammation within the gastrointestinal tract, followed by marked alterations of several digestive functions [1]. These diseases display an exasperated immune response followed by abnormal intestinal secretion with marked alterations in the patterns of motility and visceral sensation, which all together lead to abdominal pain, cramping, fecal urgency and/or constipation [2].

Although the pathogenic mechanisms underlying IBDs are scarcely characterized, available data suggests that redundant interactions between immune and non-immune cells, such as neuronal and smooth muscle cells, are critical events leading to relevant changes in the homeostasis and morphological features of the enteric neuromuscular compartments [3]. However, most of the mechanisms, through which intestinal inflammation affects the enteric neurotransmission and bowel neuromuscular functions, remain scarcely understood.

Several lines of evidence support a pivotal role of purinergic signalling in the physiological regulation of digestive functions [3] as well as in the modulation of immune/inflammatory cell activity [4], [5]. Over the last years, increasing attention has been focused on the purinergic P2X7 receptor, an ATP-gated Ca^2+^ and Na^+^ channel, characterized by low affinity for ATP, widely expressed on several immune cells (macrophages, lymphocytes, and microglia) [6]. In particular, this receptor, acting as a danger sensor in the immune system, regulates immune cell proliferation, differentiation and death [7], participating also to the fine tuning of several proinflammatory cytokine release [interleukin-1 β (IL-1β), interleukin-6 (IL-6), interleukin-18 (IL-18) and tumor necrosis factor (TNF)] [8], [9].

At present, despite evidence describing a physiological expression of P2X7 receptors in the colonic neuromuscular compartment [10], only studies, aimed at investigating the significance of these receptors in the pathophysiology of intestinal inflammation, have been performed. Recently, a pioneeristic study by Gulbransen *et al.* [11] evaluated the involvement of neuronal P2X7 receptors in neurodegeneration associated with experimental colitis. Interestingly, this work demonstrated the significance of these receptors in mediating the process of enteric neuronal death, through the activation of a complex signalling, including also the pannexin-1 channel [11]. This interesting observation suggests a critical involvement of P2X7 receptors in neuronal rearrangement occurring in the presence of bowel inflammation, thus encouraging the performance of functional studies aimed at evaluating the possible involvement of these receptors in the pathophysiology of intestinal motor dysfunctions. Based on this background, the present study was designed to investigate the expression of P2X7 receptors in the neuromuscular compartment of rat colon and to characterize their functional role in the control of colonic motility in the presence of experimentally colitis.

## Materials and Methods

### Animals

Albino male Sprague-Dawley rats, 200–250 g body weight, were used throughout the study. The animals were fed standard laboratory chow and tap water *ad libitum* and were not employed for at least one week after their delivery to the laboratory. They were housed, three in a cage, in temperature-controlled rooms on a 12 h light cycle at 22–24°C and 50–60% humidity. All experimental protocols were approved by the Animal Care and Use Committee of the University of Pisa, and were in compliance with the national and European guidelines for handling and use of experimental animals.

### Induction and assessment of colitis

Colitis was induced as described by Antonioli *et al.* [12]. Animals were anaesthetized with isoflurane and 30 mg of 2,4-dinitrobenzenesulfonic acid (DNBS) in 0.25 ml of 50% ethanol were administered intrarectally with a polyethylene catheter inserted 8 cm proximal to the anus. Control animals received 0.25 ml of vehicle. Animals underwent subsequent experimental procedures 6 days after DNBS injection, in order to allow a full development of histologically evident colonic inflammation. At that time, the animals were euthanized by overdose of isoflurane, and the colon was excised and processed for macroscopic damage score, recording of contractile activity, and histology, as reported below. The evaluation of inflammation severity was performed both macroscopically and histologically, in accordance with the criteria previously reported by Antonioli *et al.* [13]. The macroscopic criteria were: presence of adhesions between colon and other intra-abdominal organs; consistency of colonic faecal material (indirect marker of diarrhoea); thickening of colonic wall; presence and extension of hyperaemia and macroscopic mucosal damage (assessed with the aid of a ruler). Microscopic evaluations were carried out by light microscopy on haematoxylin- and eosin-stained sections obtained from whole-gut specimens, taken from a region of inflamed colon immediately adjacent to the gross macroscopic damage and fixed in cold 4% neutral formalin diluted in phosphate-buffered saline (PBS). Histological criteria included: degree of mucosal architecture changes; cellular infiltration; external muscle thickening; presence of crypt abscess and goblet cell depletion. All parameters of macroscopic and histological damage were recorded and scored for each rat by two observers blinded to the treatment.

### Immunofluorescence

Immunofluorescence was performed on frozen distal colonic tissue embedded in optimal cutting temperature mounting medium and sectioned (7 µm-thick) with a cryostat-microtome, as previously described by Antonioli *et al*. [14]. Briefly, colonic sections from control and DNBS-treated rats were fixed in 4% paraformaldehyde and incubated with 0.05 M NH_4_Cl. After Tris-buffered saline wash with 0.5% bovine serum albumin, samples were permeabilized with 0.3% Triton X-100 and incubated with rabbit anti-P2X7 receptor-ATTO-488 (1∶100; Alomone labs, Jerusalem, Israel) and with either mouse biotin-labelled anti-HuC/D (1∶100; Molecular Probes, Eugene, OR, USA) or mouse monoclonal anti-glial fibrillary acidic protein (GFAP, 1∶800; Sigma-Aldrich, Milan, Italy) for 1 hour at room temperature. To evaluate immune cell infiltration in the ENS, permeabilized tissue sections were incubated with mouse biotin-labelled anti-HuC/D (1∶100; Molecular Probes, Eugene, OR, USA) and with either mouse monoclonal anti-CD68 (1∶100, Abcam, Cambridge, UK) or mouse monoclonal anti mast cell tryptase (1∶100 Abcam, Cambridge, UK) for 1 hour at room temperature. Thereafter, sections were incubated with goat anti-mouse IgG (1∶1000; Life Technologies, Milan, Italy) labelled with Alexa Fluor 555 or Alexa Fluor 488, or with streptavidin (1∶1000, Life Technologies, Milan, Italy), labelled with and Alexa Fluor 555. Nuclei were stained with TOTO-3 iodide (1∶500; Life Technologies, Milan, Italy). Negative controls were obtained by incubating sections with isotype-matched control antibodies at the same concentration as primary antibody and/or pre-incubating each antibody with the corresponding control peptide (final concentration as indicated by manufacture’s instructions). Images of a number of colon areas, corresponding to the longitudinal smooth muscle layer (LM), circular smooth muscle layer (CM) and myenteric ganglia (MG), were acquired with a Nikon C1 confocal microscope. All microscope settings were set to collect images below saturation and were kept constant for all images. P2X7Rs or HuC/D immunoreactivities were determined by measuring the area (number of pixels) and fluorescent intensity (average intensity of pixels) of staining from 24 images captured randomly in the colonic neuromuscular compartment from each control and inflamed tissue samples. Simultaneously, the mean intensities of P2X7 receptors or HuC/D signals were normalized to mean TOTO-3 intensities for each neurons. Fluorescence values were expressed as mean values in arbitrary fluorescence units (A.U.). Image analysis and quantification of the fluorescence intensity were performed using ImageJ software (version 1.48a).

### Recording of contractile activity

Animals were sacrificed by cervical dislocation, the abdomen was immediately opened, and the colon was removed and placed in Krebs solution. Colonic longitudinal smooth muscle preparations, of approximately 3-mm width and 20-mm length, were set up in organ baths containing Krebs solution, at 37°C, bubbled with 95% O_2_+5% CO_2_. The colonic strips were oriented along the longitudinal axis, and connected to isometric force transducers (2Biological Instruments, Besozzo, VA, Italy). A tension of 1.0 g was slowly applied to these tissues. Mechanical activity was recorded by BIOPAC MP150 (2Biological Instruments, Besozzo, VA, Italy).

Krebs solution had the following composition (mM): NaCl 113, KCl 4.7, CaCl_2_ 2.5, KH_2_PO_4_ 1.2, MgSO_4_ 1.2, NaHCO_3_ 25, glucose 11.5 (pH 7.4±0.1). Each colonic preparation was allowed to equilibrate for at least 30 min, with intervening washings at 10-min intervals. A pair of coaxial platinum electrodes was positioned at a distance of 10 mm from the longitudinal axis of each preparation to deliver transmural electrical stimulation (ES) by a BM-ST6 stimulator (Biomedica Mangoni, Pisa, Italy). Electrical stimuli were applied as follows: i) 10-s single trains (sES), consisting of square wave pulses (0.5 ms, 30 mA, 10 Hz); ii) recurrent trains (rES) of square wave pulses (0.5 ms, 30 mA, 10 Hz) applied for 5 s every 60 s. In addition, we performed a set of experiments using stimulation with a submaximal concentration (1 µM) of carbachol in the presence of tetrodotoxin.

### Design of experiments

In the first set of experiments, the effects of *N*-cyano-*N*”-[(1*S*)-1-phenylethyl]-*N*’-5-quinolinyl-guanidine (A804598, P2X7 receptor antagonist, 0.001–100 µM) were assayed on sES-induced motor responses of colonic preparations maintained in standard Krebs solution.

In the second set of experiments, A804598 (0.1 µM) was tested, in the presence of apyrase (the enzyme responsible for ATP catabolism, 10 U/ml) on colonic contractions evoked by electrical stimuli, in order to provide in our model a direct evidence that P2X7 recptors are tonically achieved by endogenous ATP.

In the third series of experiments, the effects of A804598 (0.1 µM) were tested on sES-evoked contractions in colonic preparations maintained in Krebs solution containing guanethidine (adrenergic blocker, 10 µM), L-732,138 (NK_1_ receptor antagonist, 10 µM), GR-159897 (NK_2_ receptor antagonist 1 µM) and SB-218795 (NK_3_ receptor antagonist, 1 µM), in order to prevent activation of adrenergic and tachykinergic pathways.

The fourth set of experiments was designed to assay the effects of A804598 (0.1 µM) on contractile responses elicited by sES directed mainly to excitatory cholinergic nerves. Therefore, to prevent non-cholinergic motor responses, colonic preparations were maintained in Krebs solution containing guanethidine, L-732,138, GR-159897, SB-218795 and N^ω^-propyl-L-arginine (NPA; 0.01 µM), a selective inhibitor of neuronal nitric oxide synthase (nNOS).

In the fifth series, A804598 (0.001–100 µM) was assayed on cholinergic contractions elicited by direct pharmacological activation of muscarinic receptors located on smooth muscle cells. For this purpose, colonic preparations were maintained in Krebs solution containing tetrodotoxin (1 µM) and stimulated with carbachol (1 µM).

In the sixth series of experiments, the effects of 2′(3′)-*O*-(4-benzoylbenzoyl)adenosine-5′-triphosphate (BzATP, P2X7 receptor agonist; 0.01–100 µM) was tested on rES-induced contractions in the absence or in the presence of A804598 (0.1 µM).

The last set of experiments was performed to evaluate the effects of BzATP (0.01–100 µM) on cholinergic contractions elicited by carbachol (1 µM). For this purpose, colonic tissues were maintained in Krebs solution containing tetrodotoxin (1 µM).

The effects of test drugs were expressed as percent changes of control contractions elicited by ES or carbachol. The apparent potency of the P2X7 receptor agonist was expressed as EC_50_ (concentration of the agonist that produces 50% of its own maximal response). The percent maximum inhibition of control motor responses (E_max_) was also estimated. Both parameters were calculated from concentration-response curves and then averaged.

### Evaluation of ATP release

The evaluation of ATP release was performed as previously reported by Vieira *et al*. [15]. Briefly, the experiments were performed in organ baths containing standard Krebs solution at 37°C, bubbled with 95% O_2_+5% CO_2_ in the absence or in the presence of apyrase (10 U/ml) [16]. Longitudinal muscle preparations were allowed to equilibrate in the medium for at least 30 min, with intervening washings at 10-min intervals. Following the equilibration period, colonic tissues were electrically stimulated using 10-s single trains (sES), consisting of square wave pulses (0.5 ms, 30 mA, 10 Hz). Before and after each electrical stimulation, 200 µl aliquots of incubation medium were collected, frozen in liquid nitrogen and stored at −20°C. ATP was assayed by means of a luciferine-luciferase ATP Determination Kit (Roche Diagnostics Mannheim, Germany), and luminescence was determined by means of a multidetection microplate reader. The release of ATP was calculated by subtracting the basal release, measured in the medium sample collected before electrical stimulation, from the total release of adenine nucleotides, as determined after stimulation.

### Drugs and reagents

Atropine sulphate, guanethidine monosulphate, carbachol chloride, 2,4-dinitrobenzenesulfonic acid (DNBS), apyrase, were purchased from Sigma Chemicals Co. (St. Louis, Mo, USA). Tetrodotoxin, N-acetyl-L-tryptophan 3,5-bis(trifluoromethyl)benzyl ester (L-732,138), 5-fluoro-3-[2-[4-methoxy-4-[[(R)-phenylsulphinyl]methyl]-1-piperidinyl]ethyl]-1H-indole (GR-159897), (R)-[[2-phenyl-4-quinolinyl)carbonyl]amino]-methyl ester benzeneacetic acid (SB-218795), and N^ω^-propyl-L-arginine (NPA) were obtained from Tocris (Bristol, UK). Isoflurane was purchased from Abbott (Roma, Italy). Random hexamers, Moloney murine leukaemia virus (MMLV)-reverse transcriptase, *Taq* polymerase and dNTP mixture, dithiothreitol were purchased from Promega (Madison, WI). P2X7 receptor ligands were dissolved in dimethyl sulphoxide, and further dilutions were made with saline solution. Dimethyl sulphoxide concentration in organ bath never exceed 0.5%.

### Statistical Analysis

Data are expressed as mean±SEM. The significance of differences was evaluated on raw data, before percentage normalization, by performing unpaired Student’s t-tests or by one-way ANOVA followed by post hoc Dunnett’s test. P<0.05 was considered significant. Colonic preparations included in each test group were obtained from distinct animals, and therefore the number of experiments refers also to the number of animals assigned to each group. Calculations and analyses were performed using GraphPad Prism 3.0 (San Diego, CA-USA).

## Results

### Assessment of colitis

At day 6 after treatment with DNBS, the distal colon appeared thickened and ulcerated with evident regions of transmural inflammation. Adhesions were often present and the bowel was occasionally dilated. Colitis was characterized by an intense granulocyte infiltrate extending throughout the mucosa and submucosa, sometimes involving the muscular layer. An increase in both macroscopic and microscopic damage score was observed (1.2±0.8 and 1. 5±0.6 in normal rats; 12±3 and 9±1.5 in DNBS-treated animals, respectively (P<0.05 vs normal rats). The immunohistochemical analysis of colonic *muscularis externa* identified the presence of both CD68^+^ macrophages and tryptase^+^ mast cells dispersed in the muscular layers and myenteric ganglia of colonic sections from DNBS-treated rats (S1 Fig.).

### Immunofluorescence

**Figure 1:**
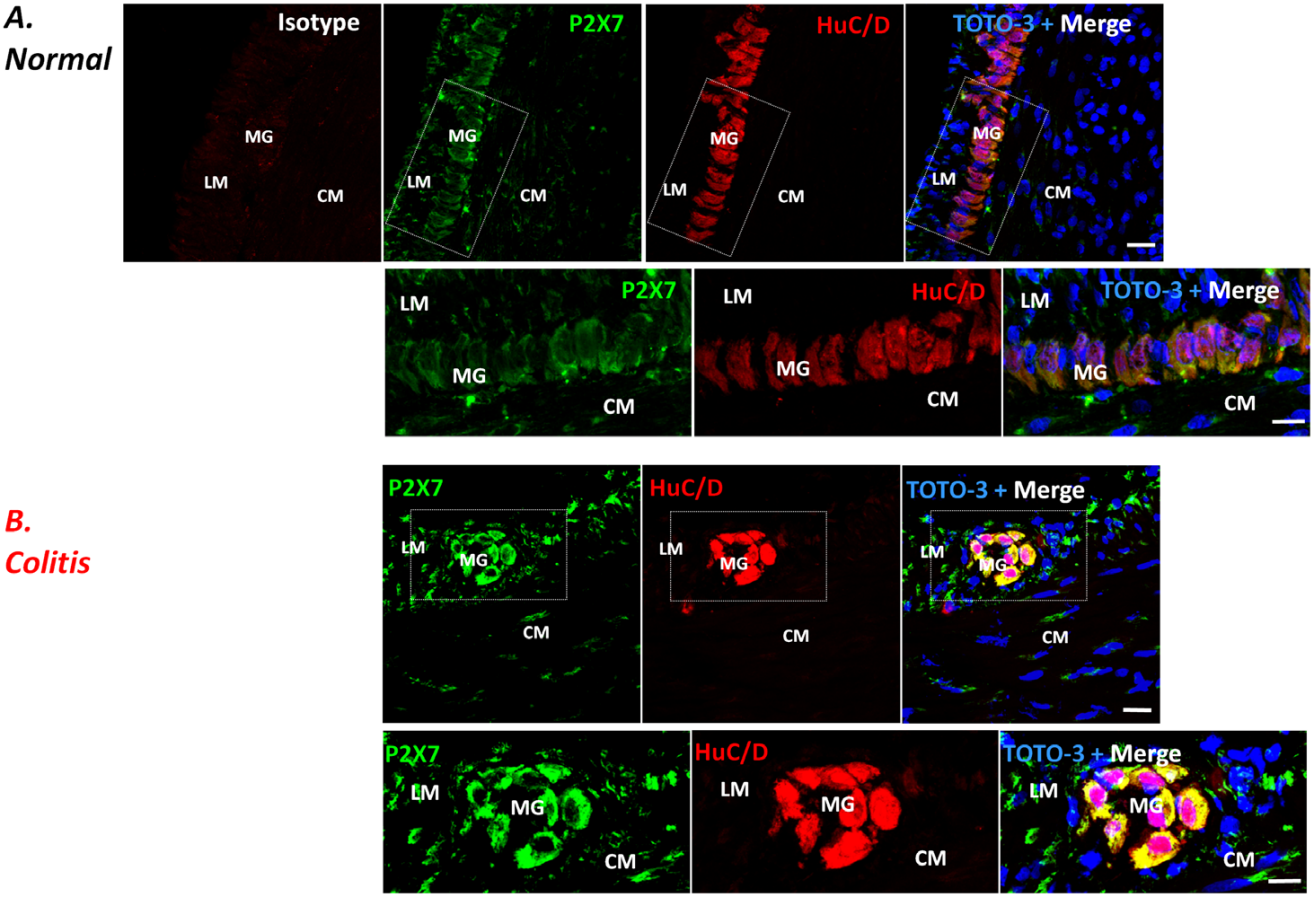
Double-staining immunohistochemistry showing the distribution of P2X7 receptors (green) and the neuronal marker HuC/D (red) in the myenteric plexus of colonic cryosections from control (A; normal) or DNBS-treated (B; colitis) rats. Nuclei were stained with TOTO-3. Scale bar: 21 µm. Enlarged view of HuC/D^+^ and P2X7^+^ cells in the myenteric ganglia of normal and colitis rats from boxed area in overlay (scale bar = 10 µm). LM, longitudinal muscle; CM, circular muscle; MG, myenteric ganglia. Isotype fluorescent image was obtained by labeling with streptavidin conjugated with Alexa Fluor 555 in presence of normal mouse antiserum instead of the primary antibody.

**Figure 2:**
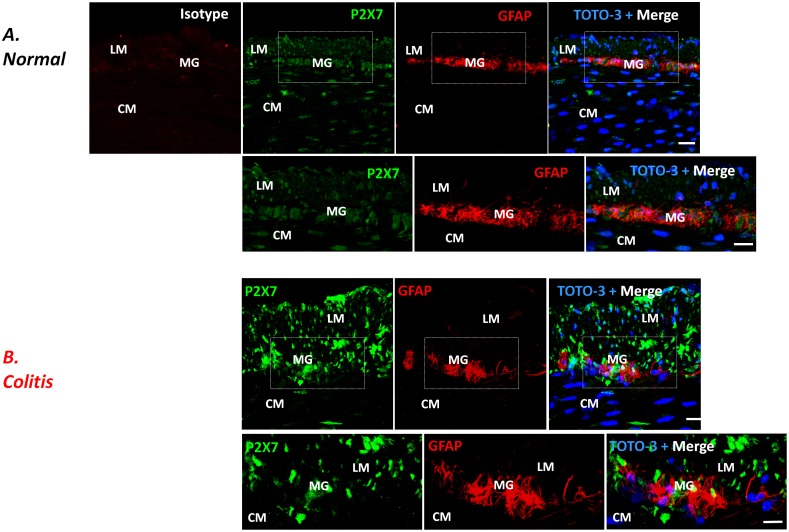
Double immunostaining showing the expression of P2X7 receptors (green) and the glial marker GFAP (red) in myenteric plexus of colonic cryosections from control (A; normal) and DNBS-treated (B; colitis) rats. Nuclei were stained with TOTO-3. Scale bar: 21 µm. Enlarged view of GFAP^+^ and P2X7^+^ cells in the myenteric ganglia of normal and colitis rats from boxed area in overlay (scale bar = 10 µm). LM, longitudinal muscle; CM, circular muscle; MG, myenteric ganglia. Isotype fluorescent image was obtained by labeling with Alexa Fluor 555 conjugated secondary antibody in presence of normal mouse antiserum instead of the primary antibody.

In the colon of control animals, the immunohistochemical analysis revealed homogenous bundles of HuC/D^+^ neurons with associated GFAP^+^ glial cells, aggregated in well-organized and detectable myenteric ganglia (Figs. 1A and 2A, and S2 Fig.). Double-label immunofluorescence of normal colon showed P2X7 receptors mainly localized in HuC/D^+^ neurons, but not in GFAP^+^ glial cells of the myenteric ganglia (Figs. 1A and 2A, and S2 Fig.).

**Figure 3:**
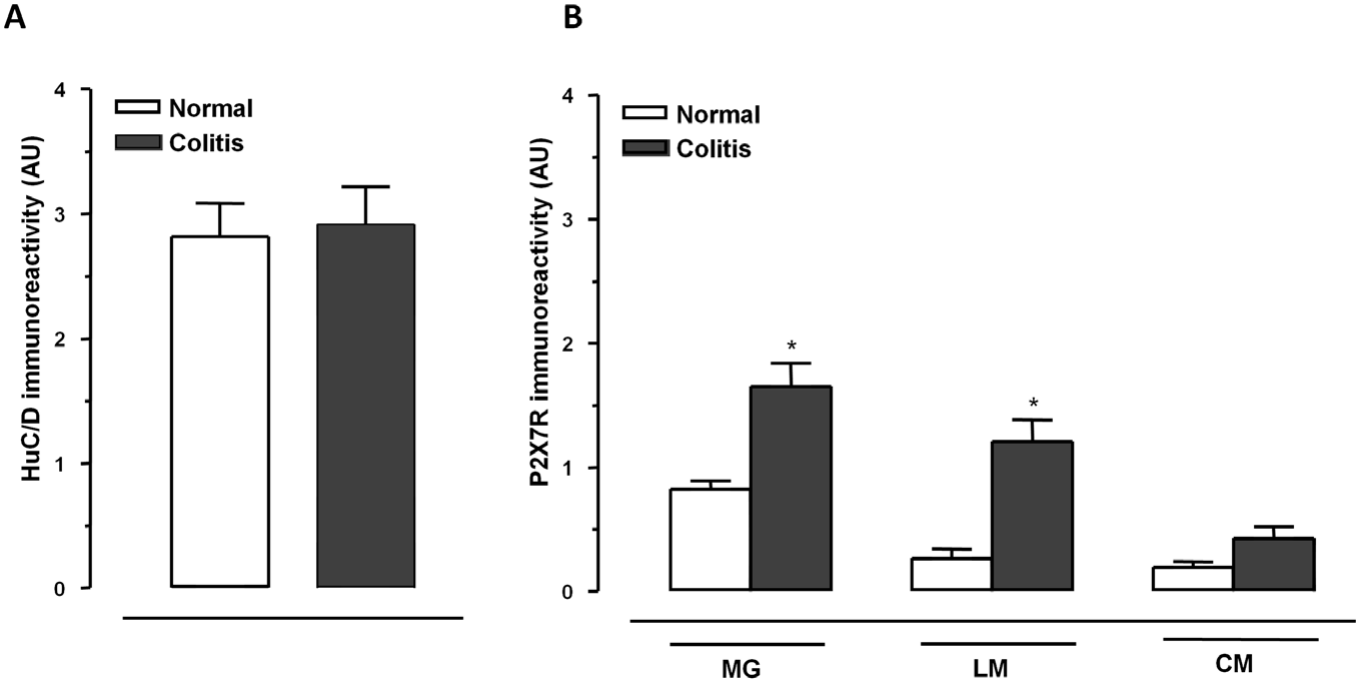
Levels of fluorescence intensity of HuC/D^+^ neurons (A) or P2X7 receptors^+^ cells (B), expressed as arbitrary units (AU), in the myenteric ganglia or the colonic neuromuscular layer (MG, myenteric ganglia; LM, longitudinal muscle; CM, circular muscle), either in the absence (normal) or in the presence of colitis, respectively. *P<0.01 versus colitis.

The induction of colitis altered the architecture of the enteric nervous system (ENS), determining loosely packed HuC/D^+^ neurons, associated with apparent variations of their cell body shape, but not of their fluorescence intensity, and dispersed clusters of GFAP^+^ glia showing distorted cellular processes (Figs. 1B and 2B, and S2 Fig.). In the presence of colitis, a rearrangement of P2X7 receptor distribution was observed in the neuromuscular layer of inflamed animals, with increased staining in myenteric ganglia, mainly at the level of longitudinal smooth muscle layer, whereas it was scanty in the circular muscle layer, as revealed by the analysis of fluorescence intensity (Fig. 3).

### Contractile activity of colonic longitudinal smooth muscle

During equilibration in standard Krebs solution, some colonic preparations developed spontaneous contractile activity, which remained stable throughout the experiment and, in most cases, was low in amplitude and did not interfere with motor responses evoked by ES or carbachol. The development of spontaneous motor activity was observed with similar frequency and amplitude in preparations from normal or inflamed colon. Electrically evoked responses consisted of phasic contractions followed, in some cases, by after-contractions of variable amplitude. Atropine (1 µM) abolished these phasic contractions, or converted them into relaxations, and only after-contractions became evident (not shown; n = 8). Tetrodotoxin (1 µM) abolished the electrically induced contractions (not shown; n = 8).

#### Effects of P2X7 receptor blockade

**Figure 4:**
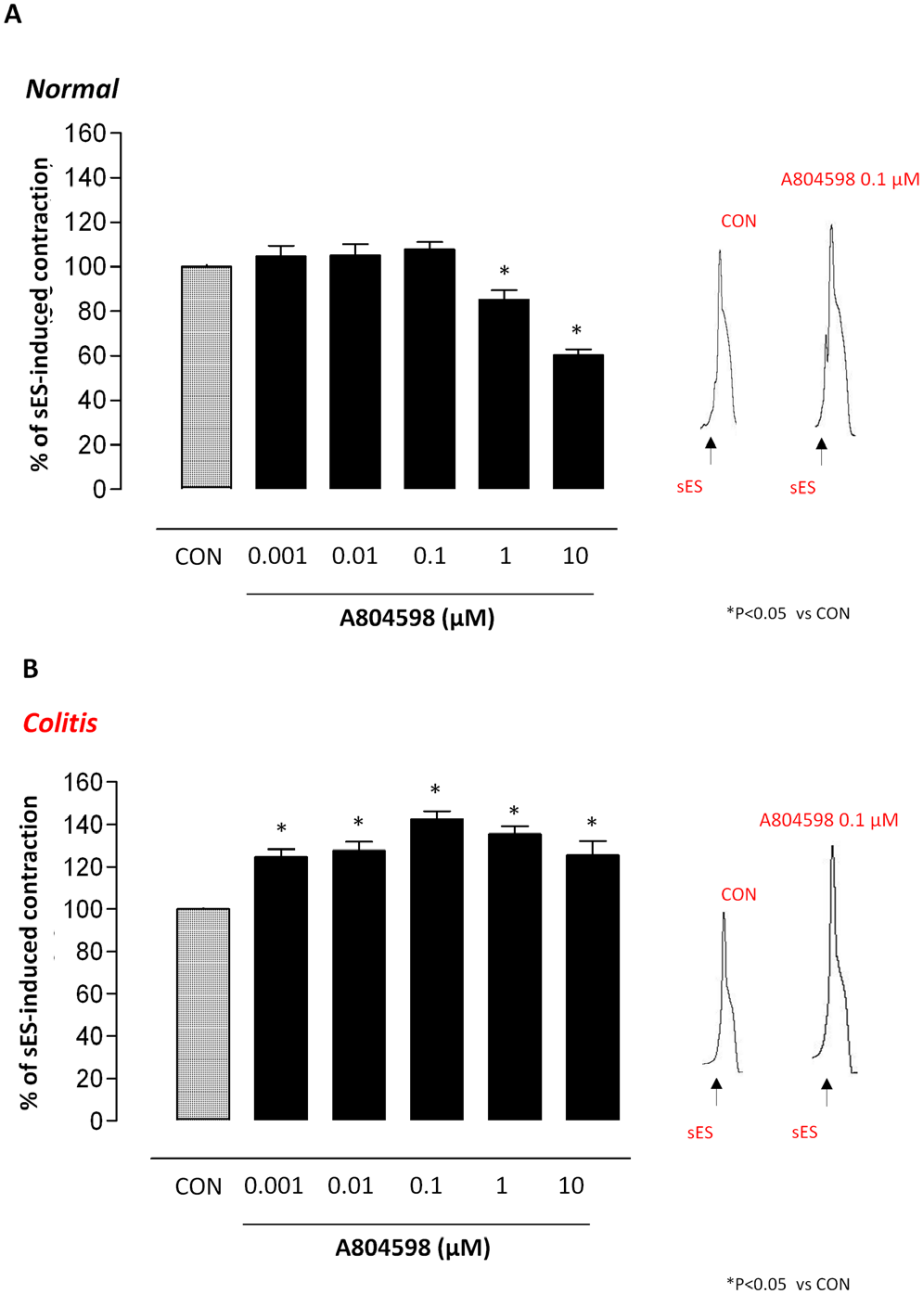
Preparations of longitudinal smooth muscle isolated from normal (A) or DNBS-treated rats (colitis) (B). Effects of increasing concentrations of A804598 (0.001–10 µM) on contractions evoked by sES (0.5 ms, 10 Hz, 30 mA, 10 s) in preparations maintained in standard Krebs solution. Each column represents the mean±SEM obtained from 6 experiments. *P<0.05, versus control (CON).

**Figure 5:**
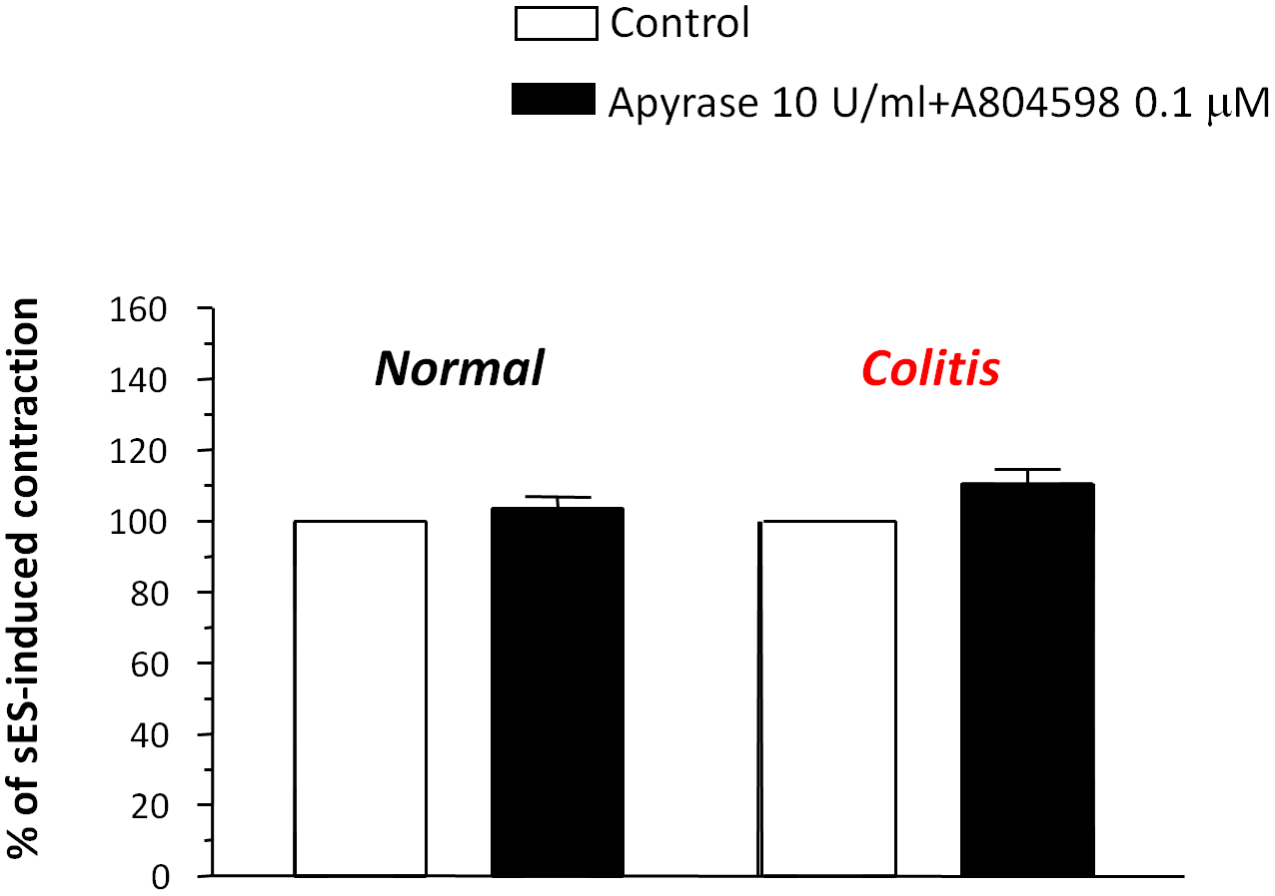
Effects of A804598 (0.01µM) on contractions evoked by sES (0.5 ms, 10 Hz, 30 mA, 10 s) in colonic longitudinal muscle preparations maintained in Krebs solution added with apyrase (10 U/ml). Each column represents the mean±SEM obtained from 6 experiments. *P<0.05, versus control.

Under resting conditions, the P2X7 receptor antagonist A804598 did not affect the spontaneous contractile activity of normal or inflamed colonic preparations (not shown). In normal colonic tissues, maintained in standard Krebs solution, A804598 (0.001–10 µM) did not affect sES-evoked contractions (+7.8±3.4% at 0.1 µM) (Fig. 4A), while in the presence of colitis, it induced a significant increase in sES-evoked motor responses, with a maximal effect occurring at 0.1 µM (+42.5±3.8%) (Fig. 4B). However, the blunting effects observed with A804598 (1–10 µM) could be ascribed to actions putatively unrelated to P2X7 receptor blockade, resulting from a loss of selectivity at high concentrations. In the presence of apyrase, the enhancing effect of A804598 (0.1 µM) no longer occurred (Fig. 5).

In colonic tissues incubated in Krebs solution containing guanethidine, L-732.138 (NK_1_ receptor antagonists, 10 µM) GR159897 (NK_2_ receptor antagonists, 1 µM), and SB218795 (NK_3_ receptor antagonists, 1 µM), sES elicited phasic contractions which were prevented by atropine and, in most cases, were converted into NPA-sensitive relaxations (not shown). Under these experimental conditions, the effects of A804598 (0.1 µM) on sES-induced contractions, in both normal and inflamed colonic tissues, were similar to those recorded in the presence of standard Krebs (normal tissues: +9.4±2.8%; inflamed tissues: +48±4%).

**Figure 6:**
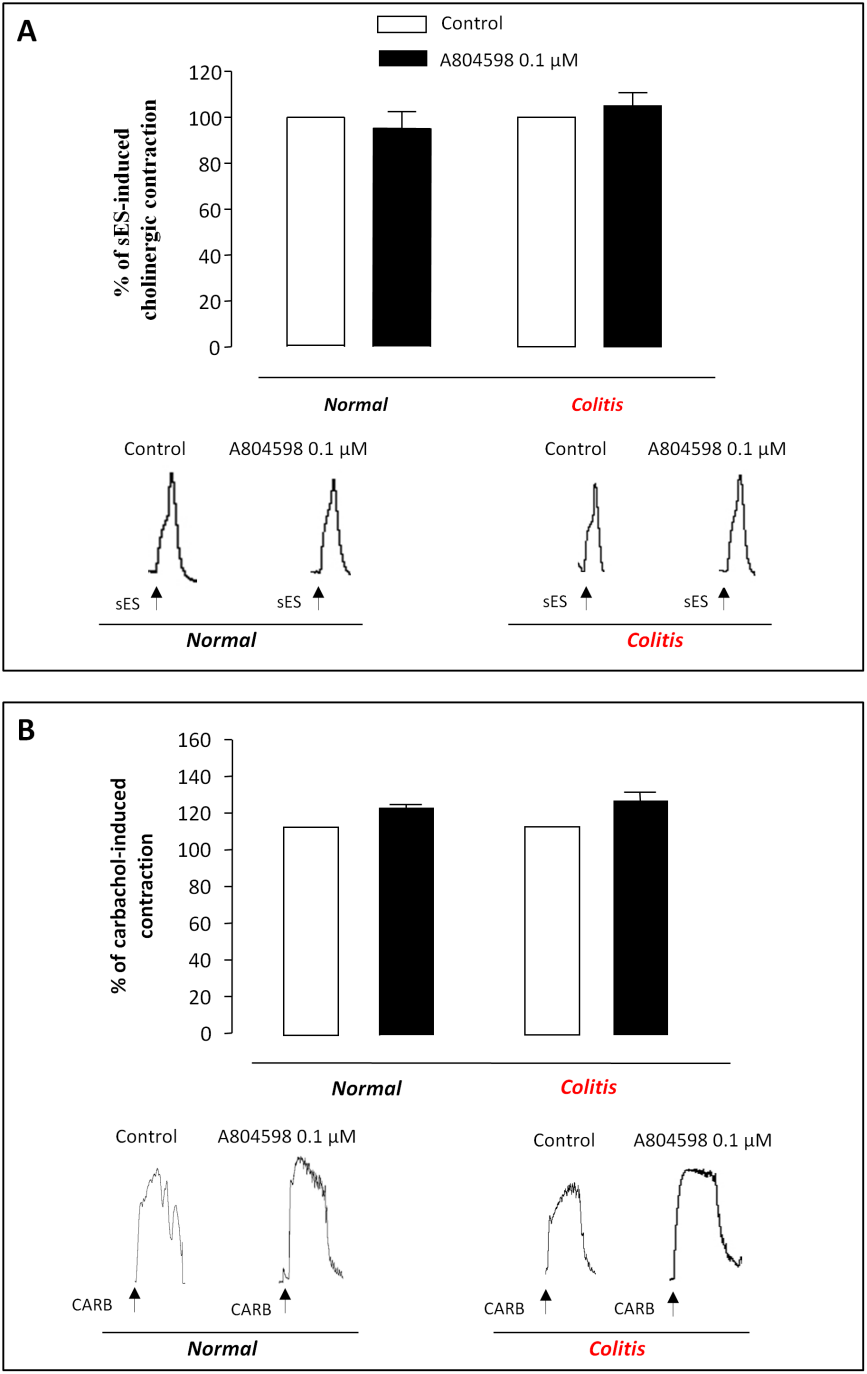
(**A**) Effects of A804598 (0.01 µM) on cholinergic contractions evoked by sES (0.5 ms, 10 Hz, 30 mA, 10 s) in colonic preparations isolated from normal or inflamed rats (colitis), maintained in Krebs solution containing guanethidine (10 µM), L-732 138 (10 µM), GR-159897 (1 µM), SB-218795 (1 µM) and NPA (0.01 µM). (**B**) Column graphs showing the effects of A804598 (0.01 µM) on contractions evoked by carbachol (1 µM) in colonic preparations isolated from normal or inflamed rats (colitis) and maintained in Krebs solution containing tetrodotoxin (1 µM). Each column represents the mean±SEM value obtained from 6 experiments. CARB, carbachol. Each column represents the mean±SEM obtained from 6 experiments. *P<0.05, versus control.

When colonic preparations were maintained in Krebs solution added with guanethidine (10 µM), NPA (inhibitor of nNOS, 0.01 µM) and NK receptor antagonists, the application of sES elicited phasic cholinergic contractions, which were largely abolished by atropine (not shown; n = 8). Under these conditions, A804598 (0.1 µM) was without significant effects both in the absence and in the presence of colonic inflammation (Fig. 6A).

In a set of experiments, the effects of P2X7 blockade were tested on contractions evoked by direct activation of muscarinic receptors on longitudinal smooth muscle. For this purpose, the effects of A804598 were evaluated on contractions elicited by carbachol (1 µM) in the presence of tetrodotoxin (1 µM). In this setting, carbachol-induced colonic contractions were not significantly affected by A804598 both in the absence or in the presence of colitis (Fig. 6B).

#### Effects of P2X7 receptor activation

**Figure 7:**
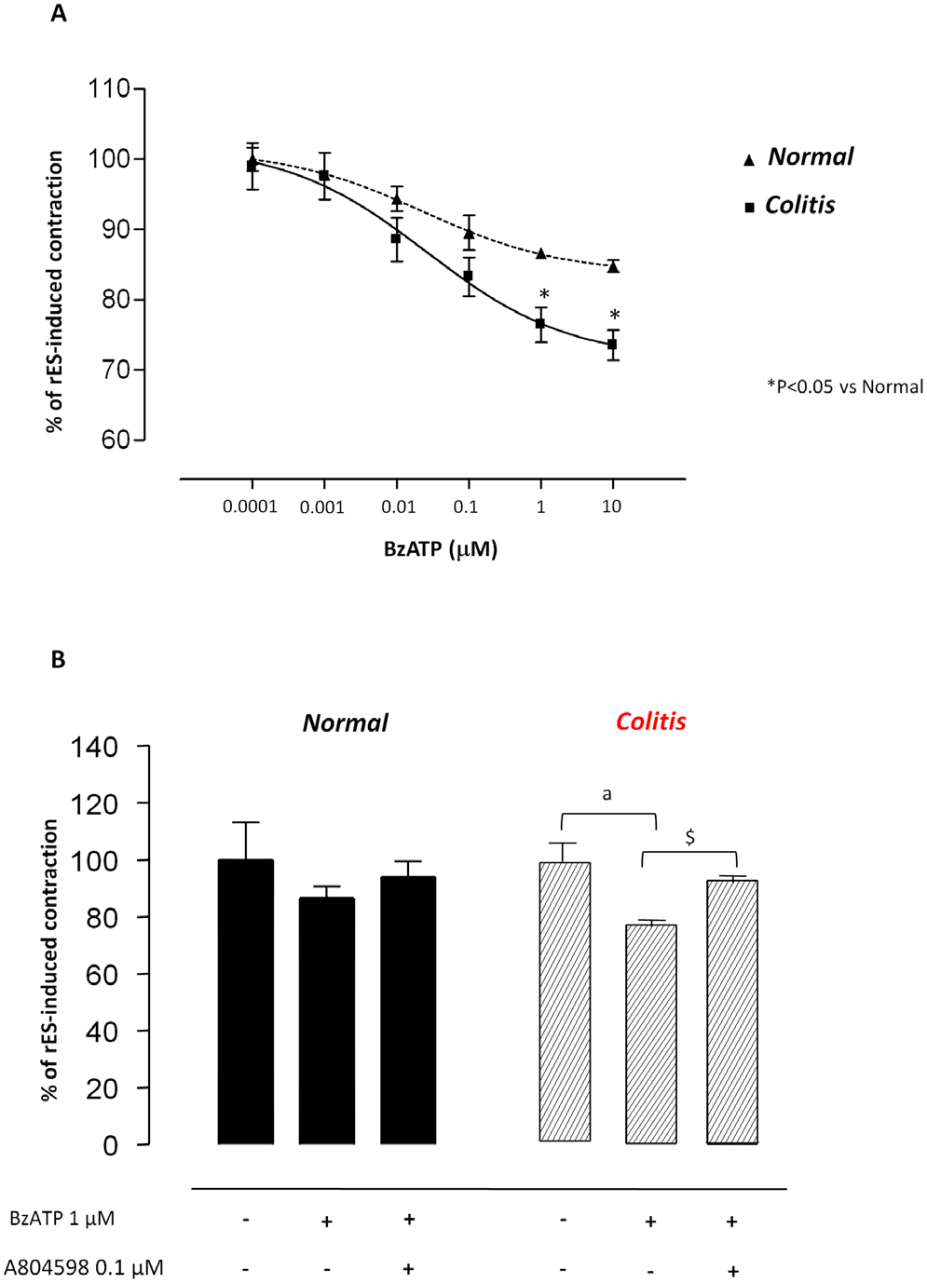
(**A**) Effects of increasing concentrations of BzATP (0.001–100 µM) on contractions evoked by rES (0.5 ms, 30 mA, 10 Hz, 5 s every 60 s) in colonic preparations maintained in Krebs solution containing guanethidine and NK receptor antagonists. (**B**) Column graphs showing the effects of BzATP (1 µM) on contractions evoked by sES (0.5 ms, 30 mA, 10 Hz), alone and in combination with A804598 (0.1 µM), in colonic preparations maintained in Krebs solution containing guanethidine and NK receptor antagonists. Each point represents the mean±SEM of eight experiments. *P<0.05, versus control.

The effects of increasing concentrations of the P2X7 receptor agonist BzATP (0.0001–10 µM) were tested on rES-induced contractions in normal and inflamed colonic preparations maintained in Krebs solution containing guanethidine and NK receptor antagonists. Under these conditions, BzATP induced a concentration-dependent relaxant response in normal tissues (EC_50_ = 23.1±3.6 nM; E_max_ = –15.2±0.8%) (Fig. 7A), with higher efficacy in preparations from inflamed colon (EC_50_ = 26.3±3.6 nM; E_max_ = –25.5±2.4%) (Fig. 7A). The inhibitory effects of BzATP were antagonized by A804598 (0.1 µM) both in normal and inflamed colonic tissues (Fig. 7B).

**Figure 8:**
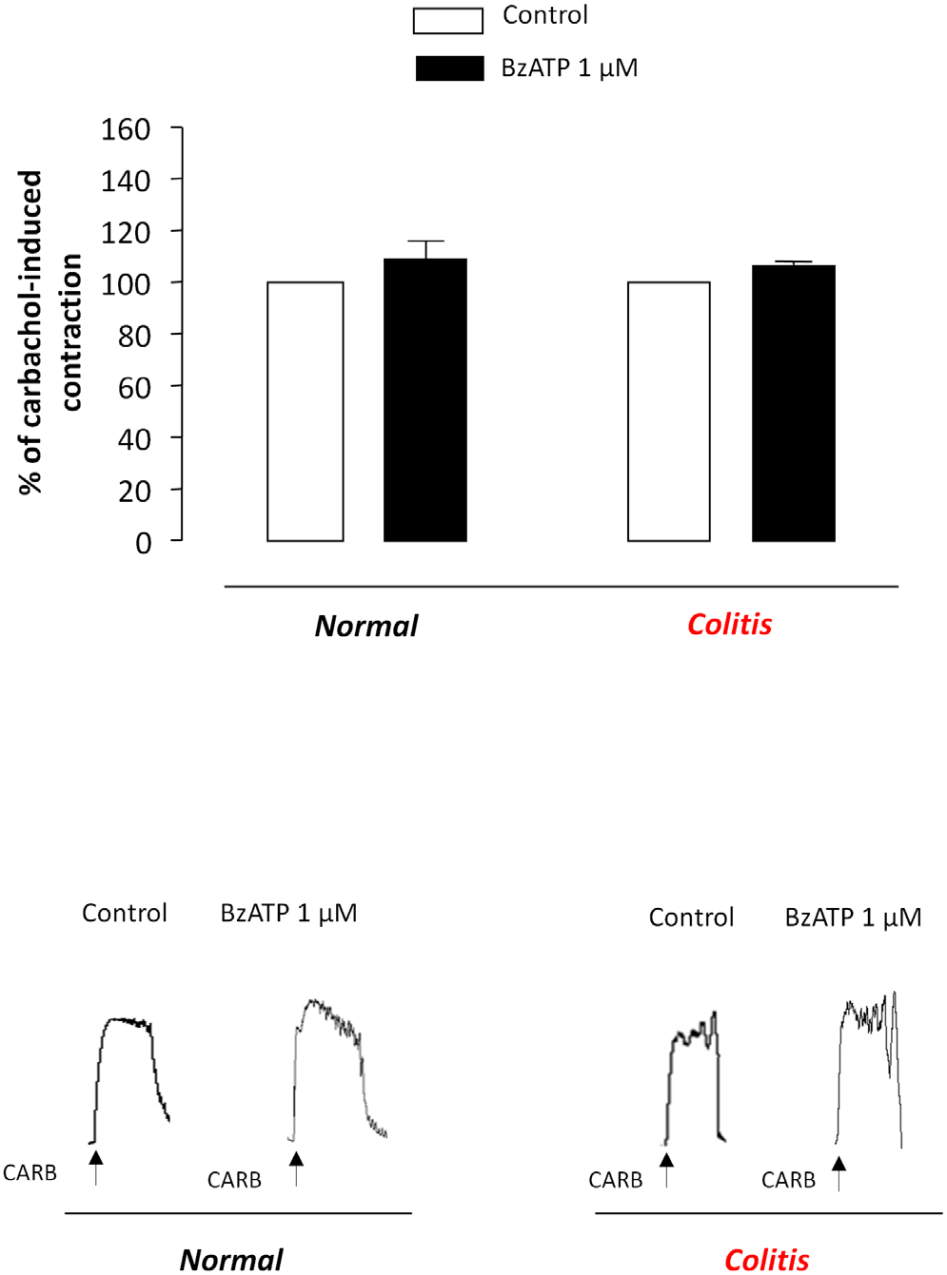
Column graphs showing the effects of BzATP (1 µM) on contractions evoked by carbachol (1 µM) in colonic preparations obtained from normal or inflamed rats (colitis) maintained in Krebs solution containing tetrodotoxin (1 µM). Each column represents the mean±SEM value obtained from eight experiments. CARB, carbachol.

In the presence of tetrodotoxin (1 µM), contractions elicited by carbachol (1 µM) were not affected by BzATP (1 µM) in colonic preparations obtained either normal or inflamed animals (Fig. 8).

### Release of ATP

**Figure 9:**
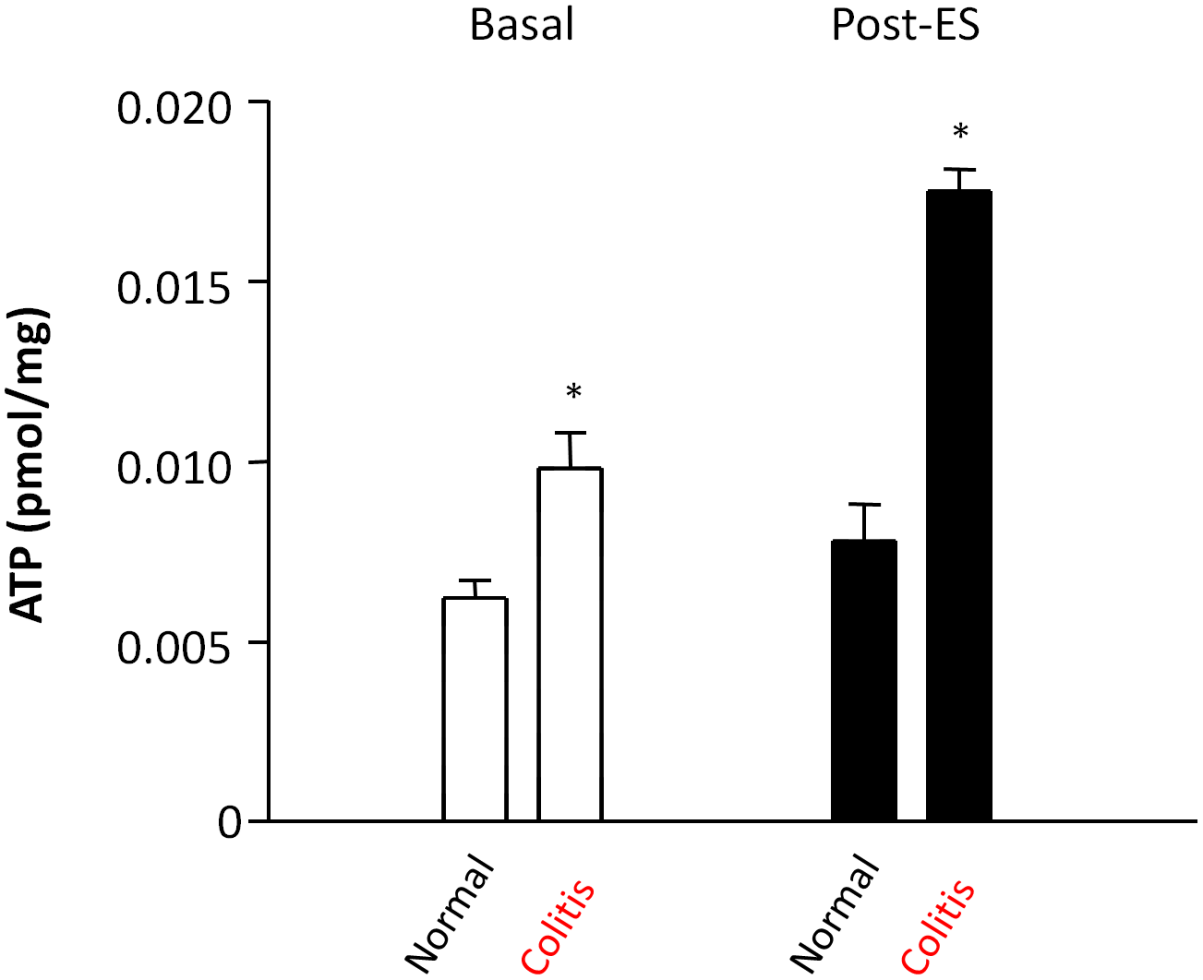
Effect of electrical field stimulation (EFS) on extracellular levels of ATP, expressed in pmol/mg of tissue, immediately before (basal) and after (post-ES) electrical stimulation of colonic longitudinal muscle preparations obtained from normal and inflamed rats. Each column represents the mean±SEM value obtained from 8 experiments. *P<0.05, versus control (CON).

Extracellular ATP concentration (pmol/mg tissue) was evaluated by means of bioluminescence assay, in aliquots of medium collected both before and after electrical stimulation of colonic longitudinal muscles preparations from control and DNBS-treated rats. A significant increase in ATP levels was observed in medium samples collected from preparation of inflamed colonic, under basal condition as compared to control (Fig. 9). Likewise, ATP levels were significantly increased after electrical stimulation in the aliquots harvested from inflamed tissues, as compared with control specimens (Fig. 9).

## Discussion

In recent years, an increasing interest has been focused on the involvement of P2X7 receptors in the pathophysiology of IBDs, where these receptors play a pivotal role in orchestrating immune cell activity as well as in regulating the neurodegenerative processes associated with gut inflammation [7], [11], [17]. Despite a physiological expression of P2X7 receptors throughout the murine [10], [11], [18], [19] or human [11] gut has been documented, data concerning the possible rearrangement and influence of these receptors on enteric dysmotility in the presence of bowel inflammation are scanty.

The present study was carried out to determine the localization of P2X7 receptors in the neuromuscular compartment of rat distal colon, as well as to characterize the possible involvement of these receptors in colonic motor disorders associated with experimental colitis. Particular care was taken to highlight the influence exerted by P2X7 receptors on the regulatory pathways of colonic neuromotility following the induction of bowel inflammation. Our findings provide evidence that: a) under normal conditions, P2X7 receptors, expressed in myenteric ganglia, take a minor part in a tonic inhibitory control on excitatory cholinergic motility, acting at neuronal level; b) the induction of colonic inflammation is associated with an increase in extracellular ATP concentrations; c) in the presence of bowel inflammation, a marked increase in P2X7 receptor immunostaining, and an enhanced modulating action of these receptors on colonic neuromotility become evident.

Under normal conditions, our immunofluorescence investigations displayed an appreciable neural localization of P2X7 receptors in the myenteric plexus. These findings are consistent with immunoreactivity for P2X7 receptors described previously throughout the myenteric plexus in guinea pigs [20], mice [11], rats [10], [18], [19] and humans [11]. We then performed functional investigations to determine the enteric neuromotor pathways regulated by P2X7 receptors. Our experiments showed that the P2X7 receptor antagonist scarcely affected the electrically-induced contractions of colonic longitudinal smooth muscle. In addition, upon incubation with the P2X7 receptor agonist, a slight concentration-dependent decrease in the amplitude of motor responses to electrical stimuli was observed. This effect was reversed by the P2X7 receptor antagonist. It was then considered that P2X7 receptor ligands might affect the evoked contractions of rat colon at neuronal and/or muscular sites, and therefore their effects were tested in the presence of a direct stimulation of colonic smooth muscle by carbachol. In this setting, the contractile responses of normal preparations were not modified by P2X7 receptor activation or blockade, thus indicating that this receptor does not operate at muscular sites to control the colonic neuromuscular functions. Taken together, these observations support the concept that, under normal conditions, P2X7 receptors are marginally involved in the modulation of rat colonic longitudinal muscle motility.

Increasing evidence highlights a critical role of ATP in the pathophysiology of inflammation [21]. In this context, special attention is being paid to the role played by P2X7 receptors in the regulation of inflammatory responses. Indeed, P2X7 receptor ligands have been tested, with encouraging results, in preclinical models of inflammation (chronic pulmonary diseases, glomerulonephritis, rheumatoid arthritis, multiple sclerosis) [22]. For this reason, the P2X7 receptor is currently viewed as an intriguing target for the development of novel therapeutic tools, as testified by a number of clinical phase I and II trials aimed at evaluating the efficacy of P2X7 receptor antagonists on several inflammatory conditions [22].

Several lines of evidence indicate the pharmacological modulation of purinergic receptors as a viable way to curb intestinal inflammation [4]. In particular, Marques *et al*. [23] demonstrated the efficacy of P2X7 receptor blockade in preventing experimental colitis, and decreasing the density of immune/in flammatory cells in the lamina propria [23]. However, a paucity of information is available about the possible significance of P2X7 receptors in the alterations of enteric neuromuscular functions associated with bowel inflammation. In order to address this issue, the second part of this study was aimed at clarifying the role of P2X7 receptors in the colonic neuromuscular activity in the presence of colitis. Our immunofluorescent analysis revealed a marked increment of P2X7 receptor distribution, particularly at level of myenteric ganglia, which occurred in concomitance with the appearance of receptor immunopositivity also in the longitudinal muscle. These findings are in keeping with recent data displaying an increase in P2X7 receptor expression in murine models of colitis [17], [23], [24] as well as in specimens of intestinal mucosa from patients with IBDs [24].

The above morphologic data are consistent with the results of our functional experiments on inflamed colon. Indeed, the blockade of P2X7 receptors elicited a more pronounced enhancing effect on electrically induced contractions, as compared to normal conditions. Of note, these enhancing effects were also appreciable after blockade of noradrenergic and tachykininergic pathways, while they were blunted upon incubation of inflamed colonic preparations with a neuronal nitric oxide synthase (NOS) inhibitor. Moreover, despite our immunofluorescence analysis revealed an induction of P2X7 receptors in the longitudinal muscular layer of inflamed colon, no significant effects were recorded when testing P2X7 receptor ligands on carbachol-induced cholinergic contractions. Taken together, these observations support the view that, in the presence of bowel inflammation, P2X7 receptors take a prominent part in the inhibitory control of rat colonic motility, modulating the cholinergic excitatory pathways through activation of inhibitory nitrergic neurons. Consistently with this proposal, a colocalization of P2X7 receptors with NOS was previously described by Hu *et al*. [20] in the myenteric plexus of guinea-pig ileum. In the same study, electrophysiological experiments revealed that the pharmacological stimulation of P2X7 receptors evoked depolarizing responses, thus supporting a modulatory role of these receptors on nitrergic neurotransmission [20]. In this context, the functional significance of the up-regulation of P2X7 receptors in the smooth muscle layers of inflamed colon remains undetermined. However, we cannot rule out the possibility that the P2X7 receptor induction in the muscular compartment of inflamed colon might fulfill regulatory activities unrelated to the cholinergic pathway or linked to other inflammatory mechanisms unrelated with enteric motor functions. To the best of our knowledge, this is the first study providing a functional characterization of P2X7 receptors in the rat colonic neuromuscular compartment in the presence of colitis. Some lines of evidence concur with the present results to suggest the involvement of P2X7 receptors in the inflammation-associated colonic motor dysfunction. In particular, Gulbransen *et al.* [11] described a tight interplay between P2X7 receptors and pannexin-1 in mediating enteric neuronal death associated with experimental colitis.

The net cellular response to purinergic stimulation is known to depend on the availability of ATP in the extracellular environment [4]. Virtually all cell types (i.e. neurons, muscular and immune cells), act as sources of extracellular ATP, via either lytic or non-lytic mechanisms (i.e exocytosis of ATP-containing vesicles, nucleotide-permeable channels, transport vesicles, and via lysosome) [4]. However, under inflammatory conditions, activated immune cells represent a relevant source of ATP [25]. In particular, this nucleotide, once released from injured and inflammatory cells through pannexin hemichannels (i.e. pannexin-1), has been shown to interact with P2X7 receptors expressed on immune cells and to activate further inflammary cells that migrate to the site of injury [26]. Based on these evidences, it is not surprising that the inflammatory microenvironment is very rich in extracellular ATP [27] particularly in close proximity of P2X7 receptors, which are known to be molecularly linked to pannexin channels [11]. In this regard, our immunofluorescence analysis confirmed the presence of infiltration by CD68^+^ macrophages and trypatse^+^ mast cells in the colonic neuromuscular layer of DNBS-treated rats. These findings are in line with data by Shi *et al*. [28], who examined the progression of TNBS–induced inflammation in colonic sections, showing a significant increase of activated macrophages in the mucosa/submucosa and muscularis externa. In line with the concept of an enhancement of extracellular ATP during inflammation [29], we observed also increased concentrations of ATP in the incubation media of colonic preparations from DNBS-treated rats both under resting conditions and after electrical stimulation. Consistently with the results by Vieira et al. [15], our ATP levels were in the range of picomolar concentration. Even though P2X7 receptors require micromolar concentrations of ATP to be activated, current knowledge suggests that ATP levels found in tissue incubation media do not likely reflect the actual ATP concentrations available for receptor activation in purinergic receptor biophase [30], [31]. There is indeed growing evidence supporting the view that, through complex interplays between different enzymes and transporters, very different levels of purinergic mediator can be maintained in the biophase of a given receptor, under different physiologic or pathologic conditions, with highly variable biologic consequences, ranging from a complete prevention of receptor activation to a highly amplified receptor stimulation. Thus, according to the novel concept of “purinome”[30], the concentration of purinergic transmitter in close proximity of a given purinergic receptor can be widely regulated to achieve low or high degrees of receptor activation, without apparent substantial changes of transmitter levels more abroad (i.e in the overall compartment of extracellular fluids).

In conclusion, the present study highlights a novel and intriguing role of P2X7 receptors in the regulation of colonic neuromuscular functions, both under normal conditions and, to a greater extent, in the presence of bowel inflammation. These observations, together with increasing knowledge about the immunomodulatory/anti-inflammatory effects resulting from P2X7 receptor modulation, might provide a promising basis for the development of novel pharmacological tools [28], potentially useful for the therapeutic management of enteric dysmotility associated with bowel inflammation. In particular, the pharmacological blockade of P2X7 receptors by selective antagonists could represent a suitable way to counteract inflammatory processes as well as for the management of those phases of IBD characterized by reduced bowel motor activity and constipation.

## Supporting Information

S1 Fig **Dual-label immunohistochemistry showing the distribution of HuC/D^+^ neurons (panels A and B), CD68^+^ macrophages (panel A) and tryptase^+^ mastcells (panel B) in the myenteric plexus of colonic cryosections from control (normal) or DNBS-treated (colitis) rats.** Scale bar = 21 µm. Isotype fluorescent image was obtained by dual labeling with Alexa Fluor 488 conjugated secondary antibody and streptavidin conjugated with Alexa Fluor 555 in presence of normal mouse antiserum instead of the primary antibodies (panel C).
(TIF)

S2 Fig **Representative image showing that preabsorption of anti-P2X7 antibody with immunogenic peptide for P2X7 totally blocks P2X7 immunoreactivity without affecting HuC/D or GFAP immunoreactivity in the myenteric plexus of colonic cryosections from control rats.** Scale bar = 21 µm.
(TIF)
