# Supplementary figures and images for: Involvement of the P2X7 Purinergic Receptor in Colonic Motor Dysfunction Associated with Bowel Inflammation in Rats
Source: PLoS One. 2014 Dec 30;9(12):e116253. doi: 10.1371/journal.pone.0116253 (PMC4280204; doi:10.1371/journal.pone.0116253)

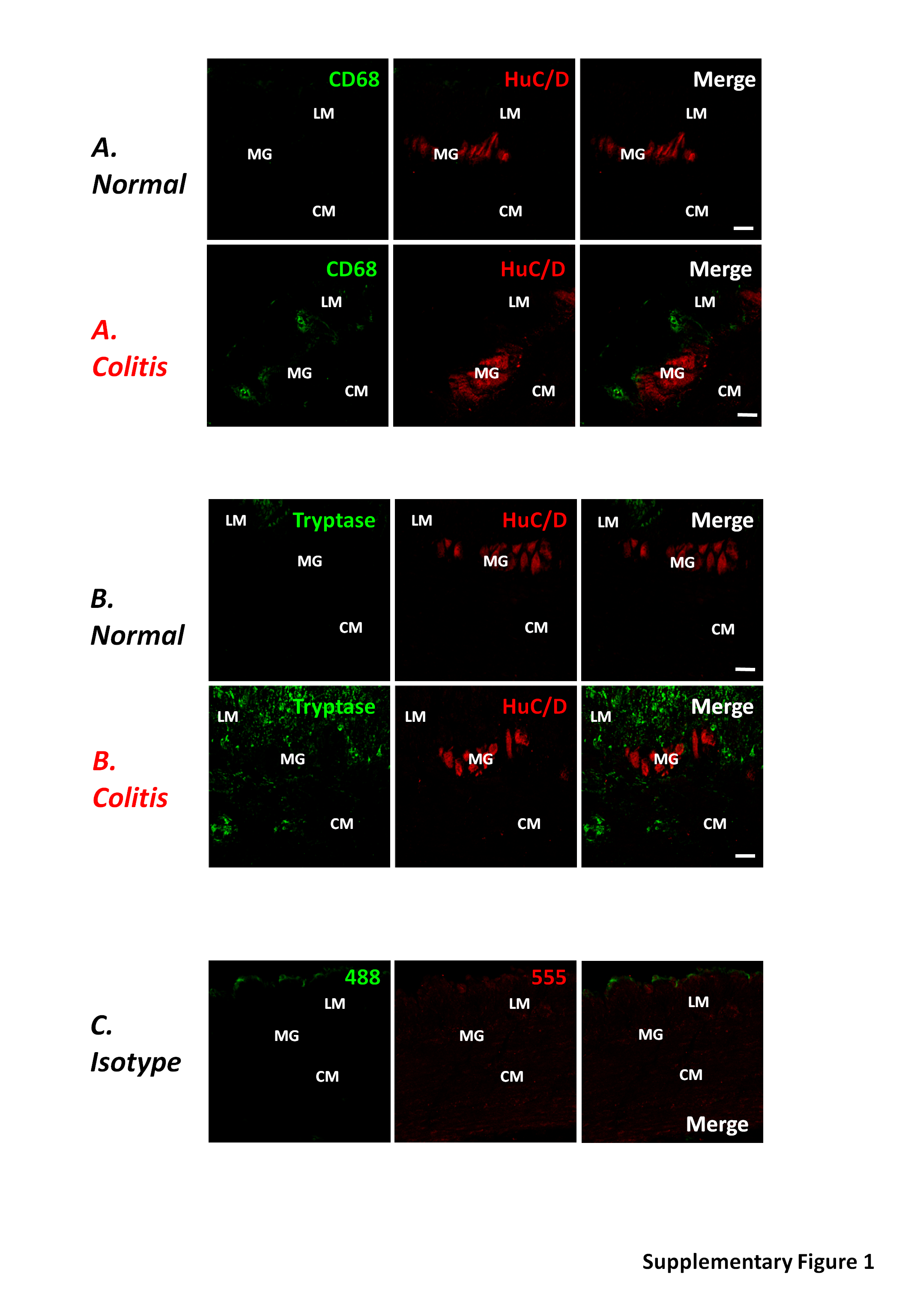

Supplement: S1 Fig — Dual-label immunohistochemistry showing the distribution of HuC/D+ neurons (panels A and B), CD68+ macrophages (panel A) and tryptase+ mastcells (panel B) in the myenteric plexus of colonic cryosections from control (normal) or DNBS-treated (colitis) rats. Scale bar = 21 µm. Isotype fluorescent image was obtained by dual labeling with Alexa Fluor 488 conjugated secondary antibody and streptavidin conjugated with Alexa Fluor 555 in presence of normal mouse antiserum instead of the primary antibodies (panel C). (TIF) [file pone.0116253.s001.tif]

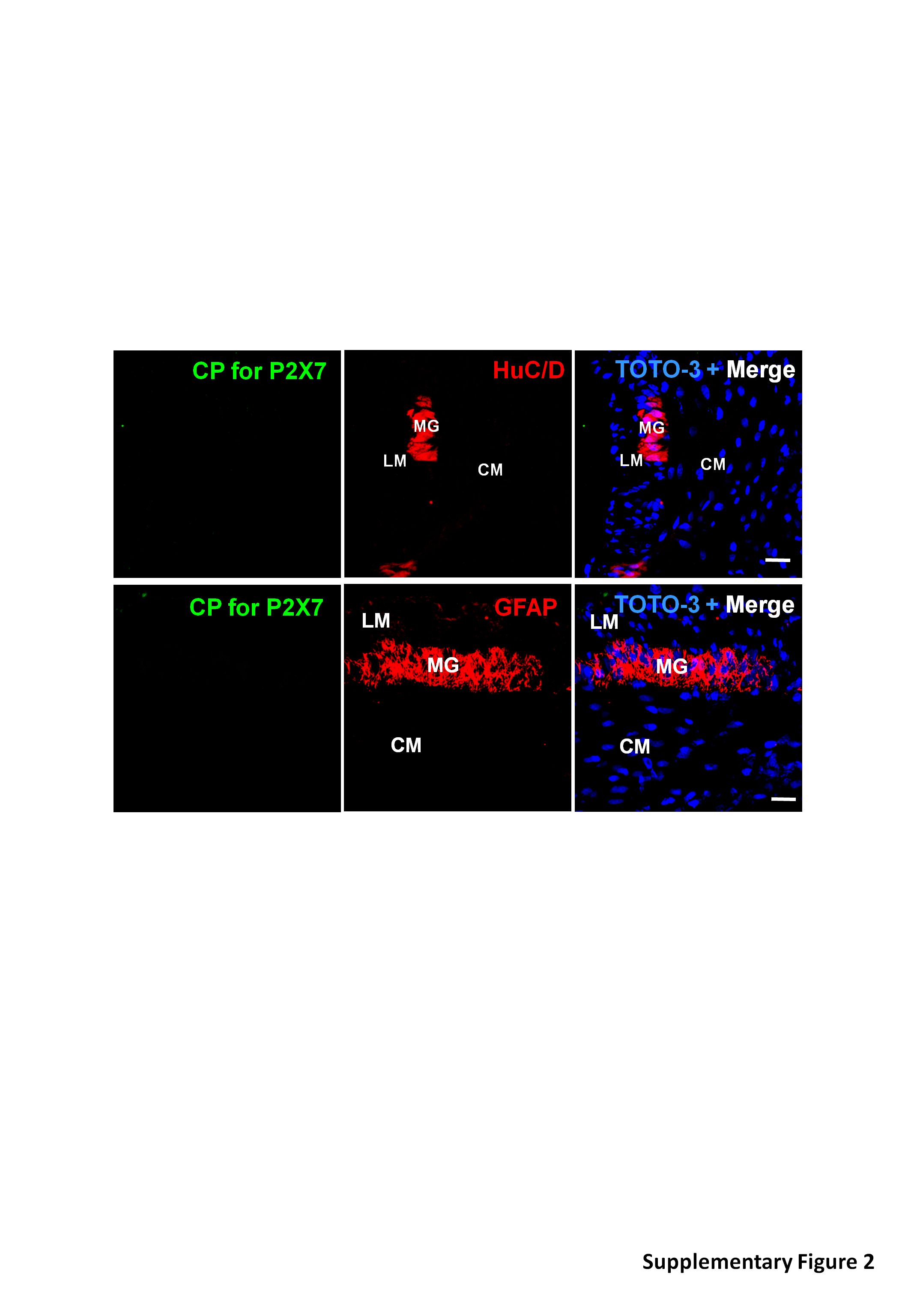

Supplement: S2 Fig — Representative image showing that preabsorption of anti-P2X7 antibody with immunogenic peptide for P2X7 totally blocks P2X7 immunoreactivity without affecting HuC/D or GFAP immunoreactivity in the myenteric plexus of colonic cryosections from control rats. Scale bar = 21 µm. (TIF) [file pone.0116253.s002.tif]
